# Redox Activity of Sodium Vanadium Oxides towards Oxidation in Na Ion Batteries

**DOI:** 10.3390/ma11061021

**Published:** 2018-06-15

**Authors:** Evan Adamczyk, Muthaiyan Gnanavel, Valerie Pralong

**Affiliations:** Normandie Univ, ENSICAEN, UNICAEN, CNRS, CRISMAT, 14000 Caen, France; evan.adamczyk@ensicaen.fr (E.A.); m.gnanavel@gmail.com (M.G.)

**Keywords:** Na_2_V_3_O_7_, sodium vanadate, Na-ion batteries, Na_4_V_2_O_7_

## Abstract

The search for new materials that could be used as electrode material for Na-ion batteries is one of the most challenging issues of today. Many transition metal oxide families as well as transition metal polyanionic frameworks have been proposed over the last five years. In this work, we report the sodium extraction from Na_2_V_3_O_7_, which is a tunnel type structure built of [V_3_O_7_]^2−^_∞_ nanotubes held by sodium ions. We report a reversible charge capacity of 80 mAh/g at 2.8 V vs. Na^+^/Na due to the V^5+^/V^4+^ redox activity. No oxygen redox activity has been observed for this material nor for the vanadium (5+) oxide Na_4_V_2_O_7_.

## 1. Introduction

Lithium-ion technology with its high energy density and long cycling life may be a solution for grid energy storage [1,2]. First introduced in commerce in 1991, Li-ion batteries now occupy a prominent place and their prominence continues to grow [3,4]. However, as the use of large-format lithium batteries becomes more widespread, the increasing demand for Lithium, combined with geographically limited reserves of minerals, is driving up prices. Therefore, sodium-ion batteries (NIBs) seem to be a viable technology for large-scale storage because of the high abundance of sodium and its attractive price. Additionally, sodium, which is the second lighter alkali metal after lithium, is one thousand times more abundant than the latter in the earth’s crust and it is geographically well distributed. Moreover, the main source of Na is sea salt where it is 60,000 times more abundant than Li. With regard to cathode materials, transition metal oxides seem very promising candidates for future Na-ion batteries applications [5,6]. Among them, the system Na-V-O has been extensively studied. By the 1980s, West et al. [7] reported the insertion of sodium in the α-V_2_O_5_ and Na_1+*x*_V_3_O_8_ lamellar materials and in the β-Na_x_V_2_O_5_ tunnel structure. These materials, and more particularly α-V_2_O_5_, were reinvestigated more recently and delivered capacities close to the theoretical capacity expected for the formation of Na_2_V_2_O_5_ based on the redox couple V^5+^/V^4+^ [8,9,10]. We also cite the work of Pereira-Ramos group on the synthesis of new sodium vanadium bronzes, which have been studied as cathode material for NIBs [11,12,13]. Recently, we explored the electrochemical and chemical reduction of a nano-structured α-V_2_O_5_ and aimed to obtain the new compound Na_3_V_2_O_5_ with a disordered rock-salt type structure [14]. In addition, we noted the reversible capacity of 150 mAh/g at an average potential of 1.8 V obtained with Na_1.5+*x*_VO_3_ [15]. The study of Na*_x_*VO_2_ material is also a topic of interest and the Delmas group were the first to study the electrochemical Na-deintercalation from NaVO_2_ [16]. This study was followed by the electrochemical characterization of both P2-Na_0.7_VO_2_ and O3-NaVO_2_ phases with first charge capacities of 100 mAh/g and 120 mAh/g, respectively [17,18].

In light of these studies, we decided to explore another sodium vanadate called the Na_2_V_3_O_7_. This phase was first synthesized by Millet et al. [19] in 1999 in order to study the properties of low-dimensional quantum magnets in the system Na-V-O. This material was the first example of transition-metal-oxide nanotubes obtained using a simple solid state synthesis without utilizing the supramolecular template mechanism. It was then deeply studied by Gavilano et al. [20,21,22] because the arrangement of vanadium magnetic moments in this compound has very particular geometrical characteristics and presents unusual magnetic properties at a low temperature. The electronic and vibrational properties of nanotubes of this transition metal oxide were also studied [23]. In this paper, we report the electrochemical de-intercalation of Na from Na_2_V_3_O_7_.

## 2. Materials and Methods

Powder of Na_2_V_3_O_7_ was obtained by using a solid state synthesis in a sealed tube from a stoichiometric amount of Na_2_O and VO_2_ (1:3 molar ratio) at 600 °C for 12 h, which was followed by a slow cooling for 6 h. The compounds were characterized by X-ray powder diffraction (XRD) using a Philips X’Pert diffractometer (Philips, Amsterdam, The Netherlands) with Bragg-Brentano geometry (CuK_α1,2_ radiation). Due to their instability in air, ex situ XRD patterns of the electrode materials were recorded using sample-holders with Kapton films. SEM images were obtained on a Carl Zeiss Supra 55 (Carl Zeiss, Oberkochen, Germany) with the In-Lens SE1 detector. The electrochemical characterization was performed in Swagelok cells with 1 M solution of NaPF_6_ in PC as the electrolyte and metallic sodium as the counter electrode. The working electrode was prepared from a mixture of active material with acetylene black in a weight ratio of 70:30. The electrochemical cells were cycled at a constant current at different galvanostatic rates on a VMP III and on a BCS-805 potentiostat/galvanostat (Biologic SA, Claix, France) at room temperature. Ex situ XRD were performed on the oxidized or reduced electrode material after being washed by acetonitrile several times to remove the electrolyte. The preparation of the Swagelok cells and washing of the electrode materials were done inside argon filled glove-box MBRAUN UNIlab Pro eco.

## 3. Results

**Figure 1:**
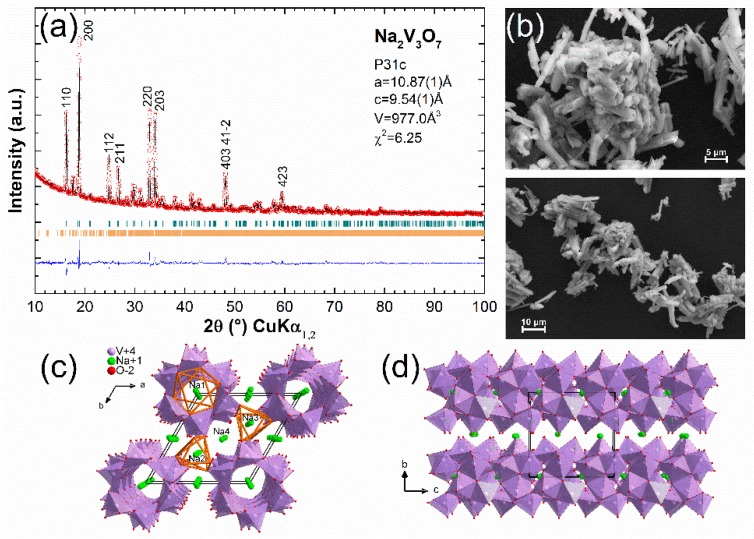
(**a**) XRD pattern and Rietveld fit for Na_2_V_3_O_7_ in cyan and η-Na_1.286_V_2_O_5_ in orange and (**b**) SEM images of the as-prepared material. Structural views (**c**) along c axis and (**d**) along a axis.

The morphology of this material consists of micro-rods with a length of between 5 µm and 20 µm, which was seen in Figure 1b. This phase crystallizes in the trigonal space group P31c with the cell parameters of a = 10.87(1) Å and c = 9.54(1) Å for a volume of 977.0 Å^3^ (Figure 1a). Therefore, η-Na_1.286_V_2_O_5_ (Na_9_V_14_O_35_) is present as secondary phase (7%). In Na_2_V_3_O_7_, square pyramids of VO_5_ are joined by edges and corners to form nanotubes along the c-axis. Sodium octahedra (Na_1_ in Figure 1c) are located inside these tubes and the other sodium polyhedra are located between two (Na_4_) or three (Na_2_ and Na_3_) vanadium nanotubes. From EDX analysis, we confirmed the ratio Na/Mn = 0.7.

**Figure 2:**
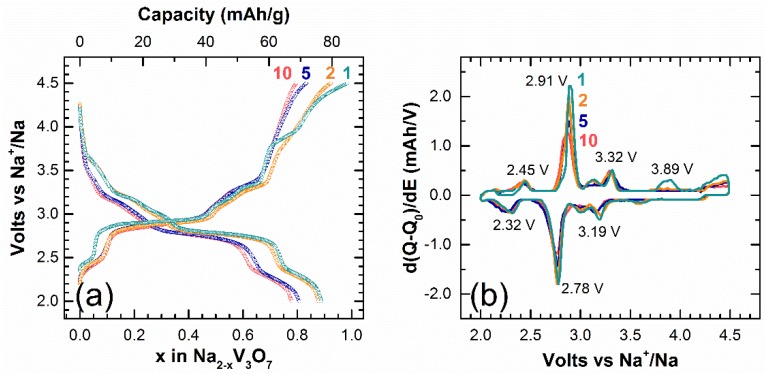
(**a**) Voltage vs. composition profiles between 4.5 V and 2.0 V at a rate of C/20 and (**b**) corresponding derivative curves. The 1st, 2nd, 5th, and 10th cycles are respectively in cyan, orange, blue, and red.

The charge-discharge curves of Na_2_V_3_O_7_ from 2 to 4.5 V vs. Na^+^/Na were performed at a rate of C/20 (1 Na^+^ in 20 h). One sodium can be extracted from Na_2_V_3_O_7_ and 0.9 Na are re-inserted, which gives specific capacities of 86 mAh/g and 77 mAh/g, respectively (Figure 2a). Multiple reversible redox phenomena can be observed on these galvanostatic cycling profiles and are also shown on the derivative curves. The main redox processes occur at 2.91 V and 2.78 V on first charge and discharge, respectively, with half width lengths of 74 mV and 77 mV (Figure 2b). Therefore, the redox process centered at 2.85 V vs. Na^+^/Na is consistent with the potential of the V^5+^/V^4+^ redox couple. A low polarization of 130 mV is observed for all the redox processes and it is reversible for the first 10 cycles. However, we note an extra oxidation peak at 3.89 V for the first charge. The charge/discharge capacities for the 2nd, 5th, and 10th cycles are 79/77, 72/70, and 69/67 mAh/g, respectively, which correspond to a capacity retention of around 80% after 10 cycles.

**Figure 3:**
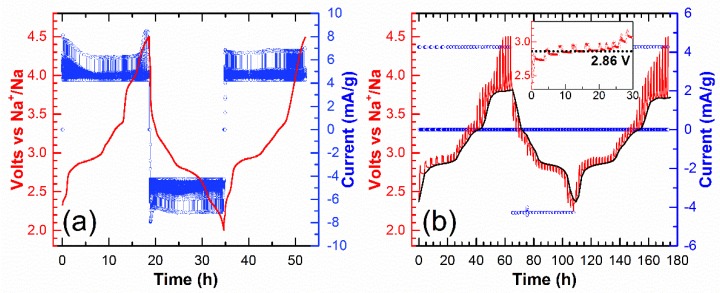
(**a**) PITT curve for one and a half cycles in the potential range 4.5–2.0 V using 10 mV potential steps in duration of 1 h and current limitation equivalent to a galvanic current *I*_lim_ = *I*_C/20_. (**b**) GITT curve with a rate of C/20 for 1 h and relaxation time of 3 h in the same potential range for one and a half cycles. Zooming in on the pseudo-plateau at 2.86 V is presented in the inset.

By performing galvanostatic and potentiostatic intermittent titration techniques on the course of the oxidation/reduction process, we can look more closely at the redox phenomena. More precisely, five domains are observed on the first charge and four domains are observed on the first discharge. For further cycles, four domains are maintained on both the oxidation and reduction cycles. These four domains correspond to the following composition ranges: 0.00 ≤ *x* ≤ 0.10, 0.10 ≤ *x* ≤ 0.45, 0.45 ≤ *x* ≤ 0.65, and 0.65 ≤ *x* ≤ 1.00 in Na_2-*x*_V_3_O_7_. The open-circuit potentials (black curve in the Figure 3b) correspond to the thermodynamic potentials for any *x* in Na_2-*x*_V_3_O_7_, which was determined by the GITT (Galvanostatic Intermittent Titration Technique). This helps highlight the four different domains. By zooming in on the second domain (0.10 ≤ *x* ≤ 0.45), a pseudo-plateau can be observed at an equilibrium potential of 2.86 V (Figure 3b). We can also see that at a higher voltage above the extra oxidation peak potential (>3.9 V), there is probably a side reaction that could explain the important relaxed potential, which is reported elsewhere [24]. Nevertheless, this side reaction is observed for the first charge only and could correspond to the difference in the first charge and discharge capacities (Figure 2). According to the PITT (Potentiostatic Intermittent Titration Technique) curve (Figure 3a), we observe only an exponential decay of the current response for every potential step, which suggests that the electrochemical process is limited by the diffusion of sodium ions. Even the pseudo-plateau at 2.86 V, which could correspond to a biphasic process, does not reveal a bell-shaped curve response in the current evolution. It is also limited by the sodium ion diffusion and not by the area of the interface between the two phases as usually encountered in a biphasic process.

**Figure 4:**
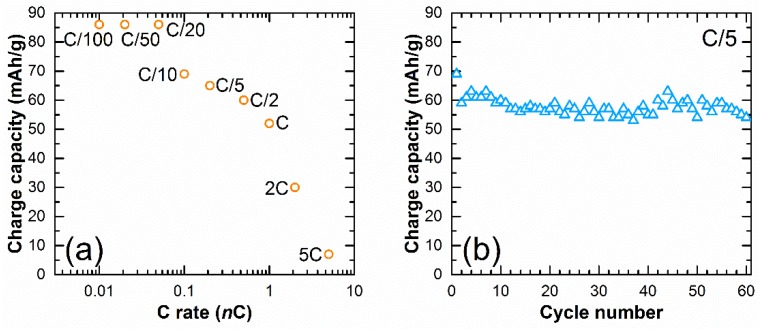
Charge capacity (**a**) vs. C rate and (**b**) vs. cycle number at a rate of C/5 in the range 4.5–2.0 V.

The same voltage-composition curves were obtained at lower rates of C/50 and C/100 without any major change in the potential of the redox processes. From the results obtained at these low rates, we show that only 1 Na can be extracted from Na_2_V_3_O_7_, which gives a maximum theoretical capacity of 86 mAh/g. When increasing the rate above C/20, the charge capacity decreases. The Figure 4a shows the capacity of first charge obtained at these different C rates. The capacity slightly decreased between C/20 and C, which gave capacities of 86 mAh/g, 69 mAh/g, 65 mAh/g, 60 mAh/g, and 52 mAh/g at C/20, C/10, C/5, C/2 and C, respectively. At higher rates of 2C and 5C, only 30 mAh/g and 7 mAh/g were achieved. The material morphology and its particle size should then be optimized for cycling at elevated rates. With more than 75% of the theoretical capacity obtained at C/5, we decided to perform the cycling test at this rate. The potential-composition curve (not shown) presents the same shape as at C/20 with the four domains of solid solution and the extra oxidation peak at 3.89 V on the first charge. However, the polarization increases from 130 mV to 250 mV. From Figure 4b, we can observe that the capacity is well maintained through cycling with 54 mAh/g on the 60th cycle. This corresponds to 84% of capacity retention.

**Figure 5:**
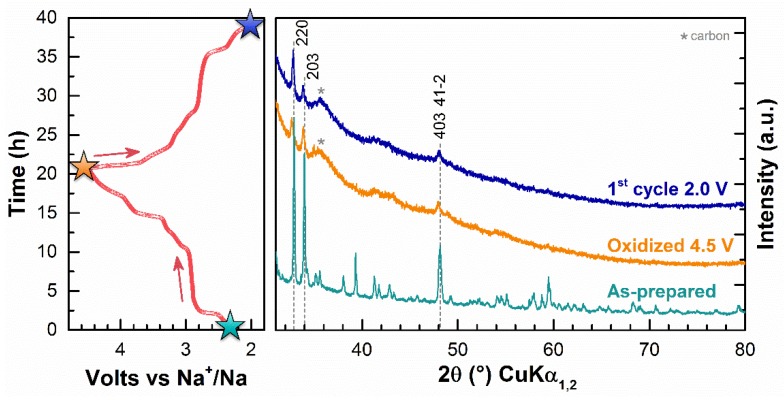
Powder X-ray diffraction patterns of the as-prepared phase and the oxidized phase at 4.5 V after one cycle between 4.5 and 2.0 V, respectively, in cyan, orange, and blue. The galvanostatic profile at C/20 is also given with the three colored stars corresponding to these three phases.

With the extraction of 1 Na from Na_2_V_3_O_7_ and due to satisfactory electrochemical reversibility, we expected the formation of a new crystalized phase of nominal formula NaV_3_O_7_. Batteries were then stopped at 4.5 V or 2.0 V on the first charge or the first discharge and characterized by ex situ X-ray diffraction (Figure 5). Starting from a well crystalized material, we obtained an almost amorphous phase on oxidation at 4.5 V after one cycle of charge/discharge. We observed from the broadening and the decrease of the intensity of the diffraction peaks that the electrode material loses its crystallinity. As seen in Figure 2 for the 10th cycle (red curve), this amorphization is accompanied by a slight smoothing of the shape of the galvanostatic curve and a broadening of the main oxidation peak on the derivative. By limiting the potential range between 3.0 V and 2.0 V in the pseudo-plateau domain, the amorphization was also observed. Afterward, we performed a chemical oxidation on Na_2_V_3_O_7_ by using NO_2_BF_4_ dissolved in acetonitrile in order to simulate the oxidized phase recovered at 4.5 V [14]. Unfortunately, the XRD pattern of this chemically oxidized phase does not correspond to the electrochemically oxidized one. The as-prepared material was partially decomposed in another Na-V-O compound: η-Na_1.286_V_2_O_5_ (Figure S1). This lamellar sodium vanadate was also present as the impurity in the as-prepared material. The chemical oxidation of Na_2_V_3_O_7_ seems to stabilize this impurity.

## 4. Discussion

Therefore, one Na can be reversibly intercalated/de-intercalated from Na_2_V_3_O_7_ at a rate of C/20, which gives the oxidized phase NaV_3_O_7_ a presumed mixed valence state for the Vanadium of V^4.33+^. However, at such high potential (>4.1 V vs. Na^+^/Na), we could expect anionic activity such as in the phase Na_2_Mn_3_O_7_. In one of our previous studies, we first demonstrate the electrochemical activity of this layered material [25]. By reducing the Mn^4+^ by inserting two sodium, we get Na_4_Mn_3_O_7_, which is another mixed valence state transition metal oxide with an average oxidation state of 3.33+ for the manganese. Very recently, this material was re-examined by de Boisse et al. [26] and this Mn-deficient layered material showed oxygen-redox activity at a potential of 4.1 V. As in the well-known Li-rich layered oxides (such as composite of Li_2_MnO_3_-LiMO_2_) [27,28,29] or in the P2-Na_2/3_[Mg_0.28_Mn_0.72_]O_2_ [30,31], the manganese Mn^4+^ cannot be electrochemically oxidized in Na_2_Mn_3_O_7_ and the redox activity at higher potential is only due to the oxygen. Meanwhile, vanadium can be oxidized into V^5+^ but an anionic activity should not be excluded. We then decided to test another sodium vanadate with an initial V^5+^: Na_4_V_2_O_7_. This material, which was synthesized by a solid state synthesis at 600 °C for 12 h in air from the stoichiometric amount of Na_2_CO_3_ and V_2_O_5_, crystalized in the monoclinic space group C2/c with the following parameters: a = 15.37(1) Å, b = 5.76(1) Å, c = 32.56(1) Å and β = 95.08(1)°. The structure is made up of isolated dimeric “V_2_O_7_” units and each unit consists of corner-sharing VO_4_ tetrahedra (Figure S2). The charge/discharge profiles of this material were obtained in the same conditions as those obtained for Na_2_V_3_O_7_ in the range 4.5–2.0 V at C/20. Figure S3 clearly shows that this material presents almost no electrochemical activity. Only 0.1 Na are extracted on the first charge and this mostly corresponds to an electrolyte decomposition with no sodium re-inserted in the electrode material. Then, no sign of anionic activity can be observed on the electrochemical curves. Even though the structures are very different between Na_2_V_3_O_7_ and Na_4_V_2_O_7_, one could assume that no anionic activity will be found in Na_2_V_3_O_7_ at a high potential. The extraction of 1 Na will lead to the formation of NaV_3_O_7_ with a mixed valence state of V^4.33+^. Looking at the XRD patterns of this oxidized phases, we could presume that the structure of the nanotubes from Na_2_V_3_O_7_, formed by the square pyramids of VO_5_, is maintained but the arrangement between these tubes has been lost. Additionally, there are no clear changes on the cell parameters but only on the shape and intensity of the diffraction peaks. This may explain the superior cyclability of Na_2_V_3_O_7_ in NIBS despite its strong amorphization. At this stage, theoretical calculations, combined with a detailed structural characterization, are necessary to determine the sodium position(s) most likely to be extracted from Na_2_V_3_O_7_.

## 5. Conclusions

In summary, we synthesized the transition-metal-oxide Na_2_V_3_O_7_, which is a tunnel type structure made of [V_3_O_7_]^2−^_∞_ nanotubes. We found that one Na can be reversibly extracted from Na_2_V_3_O_7_, which leads to a specific capacity of 86 mAh/g at an average potential of 2.8 V vs. Na^+^/Na due to the redox activity of V^5+^/V^4+^. This extraction is accompanied by an amorphization of the material. However, a good reversibility is maintained for 60 cycles at a rate of C/5. No evidence of anionic activity was observed for this material nor for the V^5+^ vanadate Na_4_V_2_O_7_. This finding confirms the interest of exploring the system Na-M-O with a transition element in order to generate new material and/or to have some better insight into the oxygen redox activity at a high potential in non-aqueous Na-ion batteries.

## Supplementary Materials

The following are available online at http://www.mdpi.com/1996-1944/11/6/1021/s1. **Figure S1**: XRD pattern and Rietveld fit of chemically oxidized Na_2_V_3_O_7_. Bragg reflections for Na_2_V_3_O_7_ and η-Na_1.286_V_2_O_5_ are indicated in cyan and orange, respectively. Figure S2: (a) XRD pattern and Le Bail fit for Na_4_V_2_O_7_. Note the presence of NaVO_3_ as impurity (14%). Bragg reflections for Na_4_V_2_O_7_ and NaVO_3_ are indicated in purple and green, respectively. (b) Structural view along the b and the a axis for Na_4_V_2_O_7_. Figure S3: (a) Voltage vs. composition profiles of Na_4_V_2_O_7_ between 4.5 V and 2.0 V at a rate of C/20 and (b) corresponding derivative curves. The 1st and 2nd cycles are, respectively, in purple and green.
