# Supplementary material for: Redox Activity of Sodium Vanadium Oxides towards Oxidation in Na Ion Batteries
Source: Materials (Basel). 2018 Jun 15;11(6):1021. doi: 10.3390/ma11061021 (PMC6024919; doi:10.3390/ma11061021)
Supplement: Supplementary file 1 [file materials-11-01021-s001.pdf]

Supplementary Material

Redox Activity of Sodium Vanadium Oxides Towards Oxidation in Na Ion Batteries

The following are the detailed information about the Supplementary Materials.

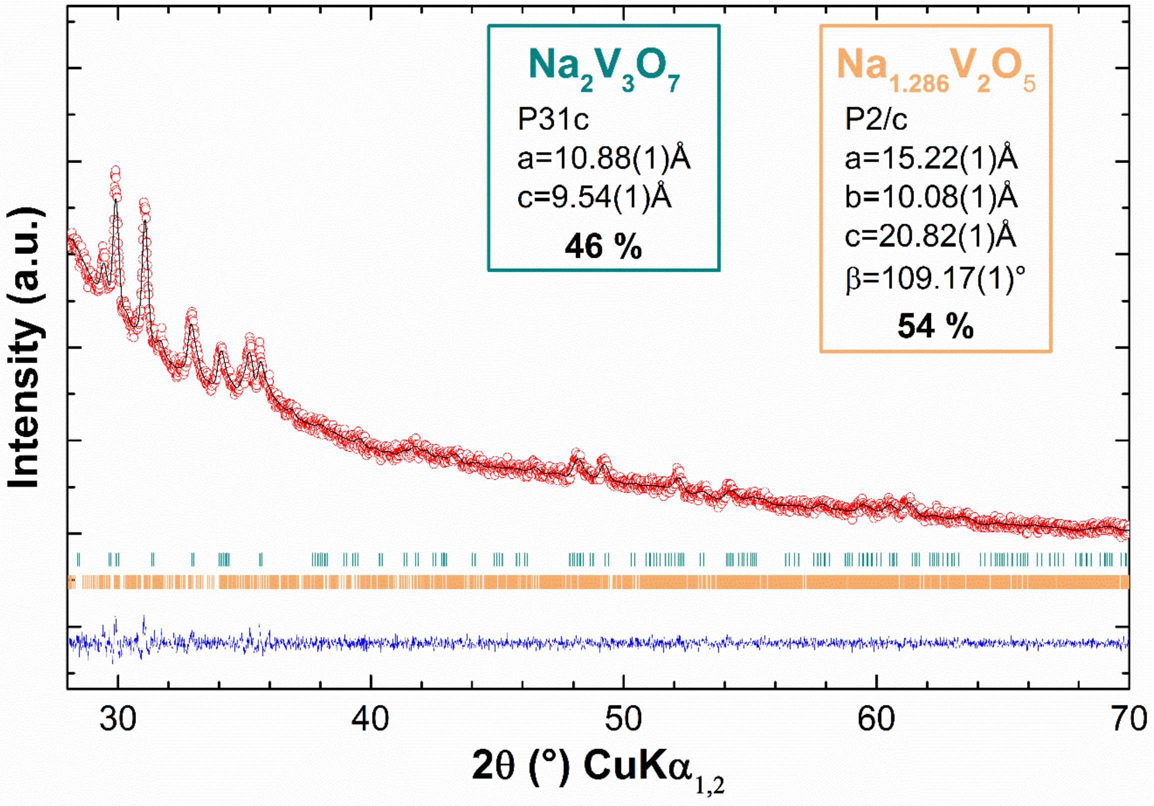

Figure S1: XRD pattern and Rietveld fit of chemically oxidized $Na_2V_3O_7$. Bragg reflections for $Na_2V_3O_7$ and η-$Na_{1.286}V_2O_5$ are indicated in cyan and orange, respectively.

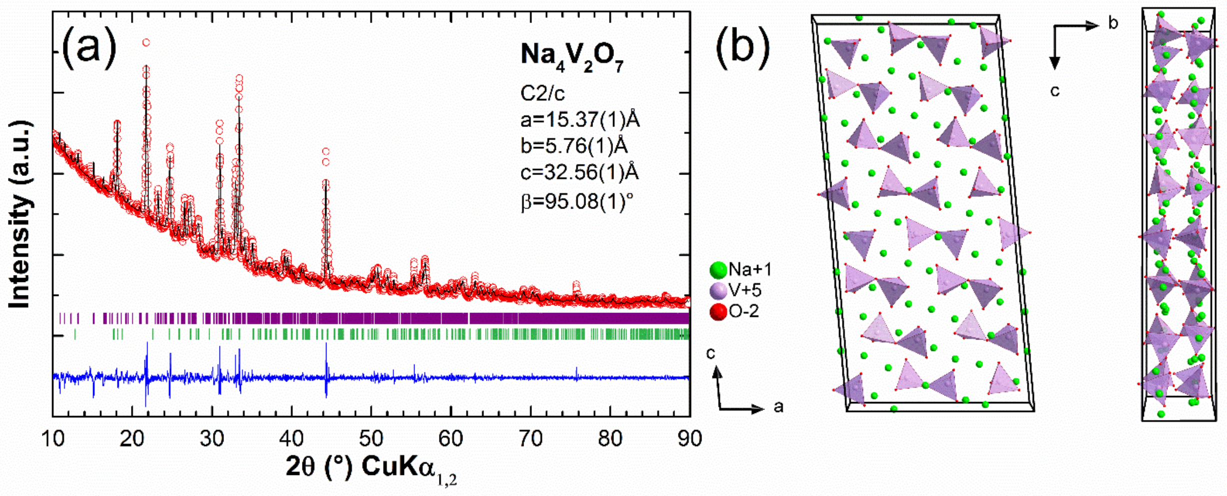

Figure S2: (a) XRD pattern and Le Bail fit for $Na_4V_2O_7$. Note the presence of $NaVO_3$ as impurity (14%). Bragg reflections for $Na_4V_2O_7$ and $NaVO_3$ are indicated in purple and green, respectively. (b) Structural view along the b and the a axis for $Na_4V_2O_7$.

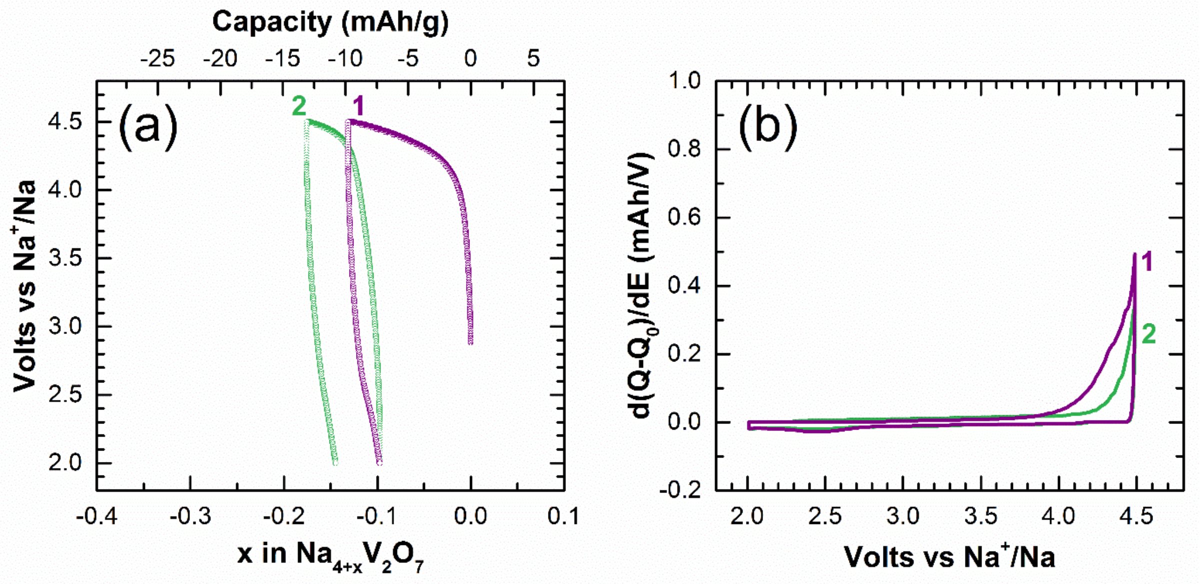

Figure S3: (a) Voltage vs. composition profiles of $Na_4V_2O_7$ between 4.5 V and 2.0 V at a rate of C/20 and (b) corresponding derivative curves.
